# The Potentials and Pitfalls of Microarrays in Neglected Tropical Diseases: A Focus on Human Filarial Infections

**DOI:** 10.3390/microarrays5030020

**Published:** 2016-08-02

**Authors:** Alexander Kwarteng, Samuel Terkper Ahuno

**Affiliations:** 1 Kumasi Centre for Collaborative Research in Tropical Medicine (KCCR), Private Mail Bag, Kwame Nkrumah University Science & Technology, KNUST, Kumasi 233, Ghana; 2 Department of Biochemistry and Biotechnology, Kwame Nkrumah University Science & Technology, KNUST, Kumasi 233, Ghana; st.ahuno@outlook.com

**Keywords:** microarray, transcriptomics, filarial, whole blood, signaling pathways

## Abstract

Data obtained from expression microarrays enables deeper understanding of the molecular signatures of infectious diseases. It provides rapid and accurate information on how infections affect the clustering of gene expression profiles, pathways and networks that are transcriptionally active during various infection states compared to conventional diagnostic methods, which primarily focus on single genes or proteins. Thus, microarray technologies offer advantages in understanding host-parasite interactions associated with filarial infections. More importantly, the use of these technologies can aid diagnostics and helps translate current genomic research into effective treatment and interventions for filarial infections. Studying immune responses via microarray following infection can yield insight into genetic pathways and networks that can have a profound influence on the development of anti-parasitic vaccines.

## 1. Introduction

Filarial infections caused by *Wuchereria bancrofti* and *Brugia* species (lymphatic filariasis (LF) and *Onchocerca volvulus* (onchocerciasis)), affect almost 200 million individuals globally. It is now evidently clear that achieving a complete elimination of the two most common human filarial infections, i.e., LF and onchocerciasis, by 2020 and 2025, respectively, requires extra effort from several stakeholders. Indeed, several field studies suggest possible resistance to one of the mainstay anti-filarial drugs, ivermectin, in some endemic communities in Ghana [1,2,3]. Hence, exploring other treatment or control strategies will be steps in the right direction. In that vein, the development of anti-filarial vaccines has been one of the top-most agenda in eliminating filarial infections. Currently, many analytical techniques exist, which can be used to elucidate the underlying molecular mechanisms during such chronic infections. These techniques range from the traditional hypothesis-driven, small-scale techniques, such as Western blots [4] and PCR [5], to the collection-driven, large-scale molecular techniques, such as microarrays [6]. Large-scale techniques, also termed high-throughput techniques, when experimentally applied can generate valuable amounts of data for genome and transcriptome studies [7]. Microarrays are specially produced, thumbnail-sized sheets of glass or silicon on which thousands of individual DNA probes have been immobilized [8,9]. These probes, being usually complementary to the target biomolecule, allow hybridization to the target biomolecule under investigation. Besides, the flexibility of probe design has generated the impetus for widespread adoption of microarray-based technologies in both industry and academic research laboratories for varied applications. The advent of microarray technology has revolutionized biomedical research [10] and has deepened researchers’ insight into host immune responses to infections [11]. In addition, it has advanced the understanding of complex and important biological quandaries such as parasite development and drug resistance, virulence, pathogenesis and the recognition of new targets for chemotherapy and vaccines [12].

### Life-Cycle of Filarial Parasites (W. bancrofti and O. volvulus)

**Figure 1:**
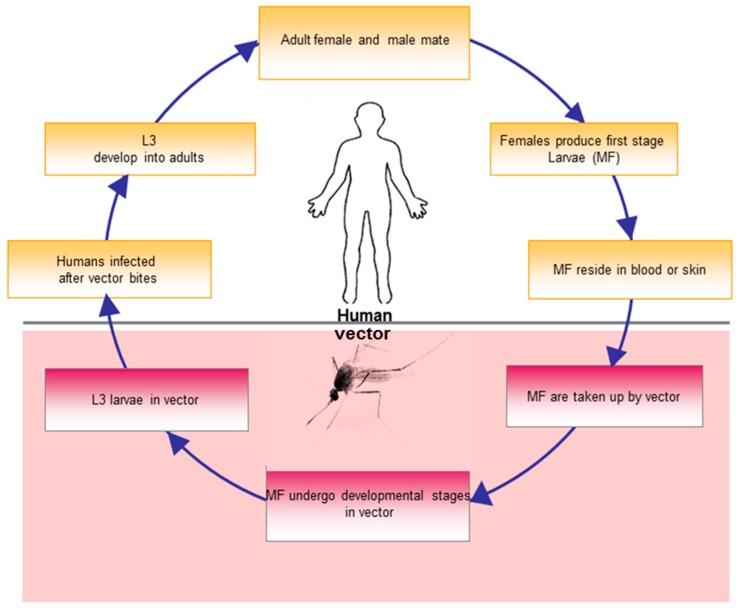
Schematic representation of the life-cycle of filarial parasites: During a blood meal, infective larvae (L3) are transmitted by vectors to the human host. L3 migrate to specific locations (lymphatic vessels, scrotal regions or dermis) where they mature, mate and produce first-stage larvae (MF) in the host. The first-stage larvae (MF) circulate in the bloodstream or skin depending on the filarial species. First-stage larvae (MF) are subsequently ingested, after which they undergo several developmental stages in the vector.

Human filarial infections are one of the debilitating vector-borne diseases affecting countries within both tropical and subtropical regions. The principal vectors for lymphatic filariasis are mosquitoes of the genera *Culex, Anopheles, Aedes* and *Mansonia* while that of *O. volvulus* is the black fly [13]. The life-cycles of filarial parasites are relatively complex with several distinct morphological stages in both vector and mammalian hosts. The early larval development of the filarial parasite occurs in the vector. However, further development and sexual reproduction have been shown to take place predominantly in the vertebrate host [14]. The life-cycle begins when an infected female vector takes a blood meal from a human host, simultaneously injecting the infective larvae (known as L3) into the dermis (Figure 1). The vector penetrates the superficial layers of the skin with its proboscis, after which the released larvae begin to migrate and develop into further larval stages and, eventually, adult worms in the body over a period of six to 12 months. In individuals with LF, mature worms reside in the afferent lymphatic vessels, scrotal regions in males or breast areas of females. In *O. volvulus*–infected patients, worms remain in the dermal regions and form nodules, termed onchocercomas. On the other hand, circulating MFs can be ingested by a feeding vector, which then matures in the gut of the vector into the infective larvae (L3) stage. The infective larvae are transmitted to another host when the infected vector feeds on that host.

## 2. Microarray Unravels Host Immune Responses during Filarial Infection

**Figure 2:**
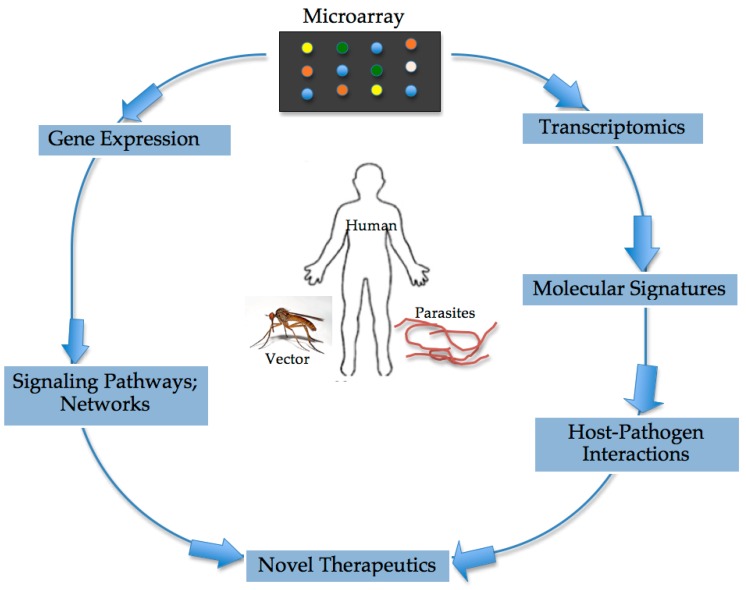
Potentials of Microarray in human filarial research.

Studying host immune responses via microarray during filarial infections can offer more insight into pathways and networks critical for the development of anti-filarial vaccines as well as towards the understanding of host-parasite interactions (Figure 2). Previous studies with different strains and gene knock-out mice have provided deeper and more reliable information on activities of the immune response in different aspects or stages of infection [15,16]. The effective elimination of infection depends on tailoring immune responses to the particular types of infection. This requires a variety of cell types and molecules that can interact coherently to generate responses needed to eliminate each type of infection. The type of response mounted by the immune system depends on several different factors or a combinations of these factors. These include but are not limited to the genetic background of the host [17], the type of strain/isolate of the pathogen [18], or the level of infection [19], age [20,21], and gender [20], among others. Beside appreciating host immune responses during infection, the microarray technology has opened up new dimensions in the diagnosis and treatment of several infectious diseases [22]. The technology provides an overview of the expression pattern of thousands of differentially regulated genes during infection. However, compared to other pathogens such as bacteria and viruses, the use of microarrays to elucidate the transcriptome of human filarial infections appears to be on the low side. Over the years, microarray has been used to identify genes previously unknown to be involved in the immune response to infection as clearly demonstrated in *Toxoplasma gondii* infection. In that study, previously unidentified genes involved in immune response were found and validated with Northern blots [23]. A recent microarray study revealed very important players involved in *Loa loa* infection, where elevated differential expression of CD8^+^ T cells in filarial-infected endemic subjects in comparison to filarial-infected expatriates were identified [24]. This suggests that there is a need to explore gene expression patterns using microarray in infections caused by other filarial nematode parasites. Such an approach will definitively deepen the current understanding of host-parasite interactions.

Furthermore, a recent study by Winter et al. [25] experimentally identified a novel member of the let-7 microRNA family bpa-miR-5364, believed to be associated with developmental transitions in filarial nematode parasites, using microarray analysis, bioinformatics and comparative genomics approaches. MicroRNAs lead to the degradation or translational repression of the target mRNA. The study by Winter and colleagues [25] demonstrated that bpa-miR-5364, among other miRNAs of the let-7 microRNA family, targets some specific mRNA, principally the Bml_27305 (regulates cell proliferation), Bml_05425 (regulates protein degradation) and Bml_25620 (regulates transcription during development). Such findings among others highlight the molecular success of microarray studies in filarial infections. The importance of miRNAs in human filarial infections is enormous, especially in the area of biomarker identification, drug targets and vaccine candidates.

Microarrays offer a comprehensive overview of gene expression, transcriptomics as well as insight into which signaling pathways and networks are affected during infections. These technologies can also be widely exploited to determine molecular signatures associated with various infections states and a more definite understanding of host-parasite interactions. Novel therapeutics can therefore be generated due to the invaluable information microarray-based technologies provide.

## 3. Prospects of Whole Blood Microarray in Filarial Infections

Filarial infections do normally elicit pathogen-specific host immune responses. These can either be systemic or localized at the infected cell. Systemic responses can be seen in altered cytokine levels as well as the host RNA phenotype in response to infection. RNA for microarray studies can be isolated either from whole blood or tissues. In the same way, it is possible to isolate from specific cells such as leukocytes for transcriptome analysis. However, RNA is susceptible to numerous factors such as the time of sample collection, transportation conditions, protectants used, pre-treatments, and extraction techniques prior to transcriptome profiling. These could affect the quality of RNA isolated and, eventually, the results of the analysis [26]. Given that transcriptome profiles from whole blood offer huge sources of information during infections, it is important to employ this approach especially when dealing with systemic infections such as LF, likewise in localized skin infections such as onchocerciasis with several infection phenotypes. While whole blood microarray technology has recently been proven to be extremely relevant in identifying several disease-associated biomarkers and hidden underlying networks, as studies in other infection models such as bacterial pathogenicity [27] and viruses [28] have suggested, much remains to be learned in the case of human filarial infections such as lymphatic filariasis and onchocerciasis.

On the other hand, it is important to highlight some frequently encountered drawbacks with whole blood microarray. A major obstacle is the relatively high proportions of globin messenger RNA present in the total RNA obtained from whole blood. Globin mRNA apparently interferes with the detection of gene transcripts [29]. In addition, some studies have reported rapid RNA degradation and transcriptomics changes after blood samples are drawn from subjects [30,31]. It is normally expected that traditional reagents such as citrate salts, heparin, and EDTA would preserve the wholesomeness of blood samples by inhibiting blood clotting, however these reagents do not stabilize mRNA transcripts [32]. A study by Debby et al. (2004) [31] observed up-regulation of genes related to hypoxia and down-regulation of genes related to metabolism and cell cycle in whole blood samples when RNA was not immediately isolated after blood collection. In addition, it has been observed that in comparison with leukocyte isolation, mRNA isolated from whole blood is normally found to be associated with increased noise and reduced gene expression [31,33] due to the heterogeneous cellular nature of blood samples. In response, several protocols for blood RNA stabilization have been developed. One is the PAXgene blood RNA system which stabilizes RNA immediately upon blood collection [34]. Fricano et al. (2011) [35] have also demonstrated in a recent study that the QIAzol-based RNA stabilization and isolation method coupled with the NuGEN Ovation Whole Blood Solution system are cost-effective and particularly suitable for transcriptomic profiling of minimal volumes of whole blood, typical of those obtained with small animal species. Other protocols to reduce globin mRNA, such as GLOBINclear (Ambion, Applied Biosystems) or globin peptide nucleic acid (PNA) oligos, are also now readily available, although such methods have been associated with some issues such as being time-consuming, low-throughput, and prone to additional experimental variability [36].

Conventionally, cell separation methods are used to extract RNA from whole blood when cell-specific microarrays need to be performed. It has been shown that following cell separation methods, α and β globin mRNA appear to be the most abundant transcripts present in the total RNA extracted from leukocyte-enriched populations [33]. Therefore, the separation method used could influence sample-to-sample variability in the microarray assays. In as much as cell separation methods lead to the removal of erythrocytes from whole blood, there are, however, compelling reasons to study gene expression from whole blood rather than from sub-populations of cells. This includes the profound ability to capture a snapshot of the expression profiles that accurately reflect the transcriptional state at the time of blood collection. Additionally, the whole blood microarray approach preferably avoids additional processing steps, which apparently induce some degree of cell activation during cell separation.

One major limitation to microarrays is that compared to other next generation sequencing (NGS) technologies such as RNA-seq, the platform cannot be used to detect unknown transcription products [37]. Another trade-off is that there is decreased sensitivity of the arrays to the detection of genes with low expression levels (low-abundance genes). Another issue of concern with microarray technology is data analysis and the extraction of biological knowledge [38]. This is due to the enormous and complex datasets generated from the microarray experiment thereby placing a high demand on analytics software and technologies, as well as scientists with high computational and bioinformatics skills [39]. However, following the recent wave in data science (https://datascience.nih.gov/) and the massive improvements in bioinformatics for transcriptome analysis [40], scientists can now draw meaningful deductions and make better inferences from the huge datasets normally associated with microarray experiments.

## 4. Appreciating the Interplay between the Filarial Parasite and Human Host: The Role of Microarray Technology

One of the fascinating features associated with filarial infections is immunosuppression. This actually reflects an evolutionary relationship between filarial parasites and their host. Currently, a comprehensive review on “cross-talk between parasites and the host immune system” has been described [41]. The global down-regulation of genes and effector molecules of the host immune system is strongly seen in several instances where heavily infected individuals even fail to mount an appropriate immune response [42], i.e., permissive to the microfilariae (MF), the transmission stage of the parasite. The immunosuppression of the host immune response is believed to be driven by both female adult worms and MF. However, immunosuppression is presumably suggested to be the female adult worms’ mediated strategy to create a suitable milieu for the survival of the baby worms, i.e., MF. Having established the co-evolutionary interaction between the filarial parasites and their host [41], identifying the molecular mechanisms and immune signatures with biological relevance will help better appreciate this long-term host-parasite interaction.

In LF infection, the well-characterized asymptomatic phenotypes include: patent infection, i.e., positive for circulating filarial antigen (CFA) and MF; latent infection, where infected individuals are positive for CFA but have no circulating MF; and, finally, the chronic pathology group, which is characterized in most cases by the absence of both CFA and MF. Understanding the drivers of the host immune response that lead to the above-mentioned phenotypes will be helpful in appreciating the host-parasite interaction. Several attempts have been made towards elucidating the underlying regulators that influence the interplay between the filarial parasites and host at the genomic level as well as functional protein levels. The development of a vaccine against human filarial infections is crucial and obviously requires several validated experimental and animal infection models. However, until recently not much was known regarding the molecular underpinnings during parasitic infection in general and filarial nematodes in particular. In order to understand the gene expression profile of the host response during filarial infection more comprehensively, a high-throughput approach is required. Given that filarial infections are chronic and the fact that adult worms can live for 10 years or more, assessing the impact of the possible confounding factors, which contribute to infection outcome, is essential. Indeed, it has been previously established that ethnicity, age, gender, and genetic background, among others, could affect the host immune response and significantly impact gene expression profiles in many tissues to a varying degree [43,44].

During filarial infection, a higher degree of variability is introduced due to the constant interaction between parasite and host. In most cases, factors that could influence gene expression patterns are the source and type of tissue used for the microarray experiment. Although microarray brings more to the table, it has to be indicated that the proximity of the tissue, for instance used as a control, to the disease tissue could have a strong impact on the gene expression profile of the control tissue. Elsewhere, normal tissues close to tumors have been shown to have genotypically altered expression profiles [45,46]. Moreover, factors such as the level of disease-associated inflammation could strongly impact the pattern of gene expression. Furthermore, other bystander effects such as secondary infection have been suggested to potentially influence the gene expression profile in patients [47]. While studying host immune responses with microarray technology is very essential in understanding the underlying molecular mechanism during filarial infections, microarray experiments, which focus on the specific nematodes, can provide insights into parasite stage-specific responses. Taken together, it is extremely important to exercise a lot of precaution on sample selection and preparation when using microarrays, particularly in human filarial infections, given the complex nature of the infection.

## 5. Capacity of Microarray to Reveal Induced Signaling Pathways and Networks during Filarial Infections

The immune system relies heavily on signal pathways to coordinate responses in a professionally efficient manner. Therefore, manipulating the signaling pathways to a greater extent has a functional consequence on the immune response and could significantly influence infection outcome. Parasite-derived molecules that interfere with signaling pathways are an interesting, intense area of investigation. Apparently, among the strategies employed by filarial parasites is interference of some signal pathways which finally leads to diverting the onslaught of the attack of the host immune responses. This could perfectly reflect the immunosuppression scenario observed in MF+ individuals, compared to latent infection where a possible set of pathways is believed to contribute to protection against MF. Although a lot of work has gone into the determination of the functional relevance of cytokines, chemokines and immunoglobulins, a lot remains to be learned at the transcriptional level on the impact of the direct interaction between the host immune cells and the filarial parasites. Given that the host immune response is primarily dependent on the integration of several signals induced by the presence of the parasites (adult worm or MF), it is critical to study the specific signaling pathways that are significantly impacted during filarial infections. Knowledge obtained from such high-throughput studies will deepen the current understanding of the host-parasite interaction and the potential identification of suitable anti-filarial vaccine candidates and biomarkers, which could eventually facilitate the control of filarial infections. In the subsequent section of this review, the roles of other microarray platforms such as protein and miRNA microarrays are discussed.

## 6. Potential of Other Microarray Platforms

It is worth knowing the diversity of microarray technologies currently applied in modern research. Advances and inventions made over the past few decades did not leave out microarray technologies. Current microarray technologies have the potential of elucidating the transcriptome and proteome of the filarial parasite. They can also bridge the gap of understanding in the host-parasite interaction during infections. Microarrays can be used in comparative genomic hybridization studies, which are a molecular cytogenetic approach for the genome-wide detection of chromosomal deletions and amplifications. Genomic hybridization has the potential of genotyping individuals for genetic differences, such as single-nucleotide polymorphisms (SNPs), which might be associated with various disease states [48].

More importantly, the question of which platform (compared to current NGS) to use mostly depends on the researchers’ goals for a particular experiment as well as being able to strike the difference between the cost and performance of the tools in question. Research questions likely to be addressed when choosing between microarrays and other technologies may not be limited to: the relevance of absolute quantitation, the need to discover novel genes of interest, or the expression level of transcripts. Indeed, one would also have to consider how to make meaningful scientific connections and conclusions from the enormous amounts of data often generated.

### 6.1. microRNA Microarrays

The discovery of disease-specific miRNAs has opened up numerous possibilities for alternative diagnostics and the identification of prognostic biomarkers of several diseases such as cancer, metabolic diseases, and viral as well as parasitic infections [49,50,51]. A growing collection of circulating miRNA of filarial origin during infections has been reported by various studies [52,53,54,55,56]. MicroRNA microarrays are innovative technologies capable of contributing knowledge to the stage-specific parasite life-cycle [57], the host-parasite molecular interaction, as well as the mechanisms of specific immune regulators [58] deployed by these parasites to evade the host immune system.

miRNA expression profiles can be used to study gene expression, developmental and evolutionary processes in parasites and the selection of therapeutic targets against most filarial nematodes [58]. Circulating miRNAs from filarial nematodes can be inhibited using single-stranded oligonucleotide analogs [59] that hybridize with specific miRNAs, thereby offsetting the modulatory effects of filarial nematodes on the host immune response. An innovative way of achieving this could also be with the use of synthetic oligonucleotides (antagomirs) [60] as silencers of circulating filarial miRNAs.

### 6.2. Functional Protein Microarray

In functional protein microarrays, the miniature reaction volumes of expensive protein reagents coupled with the powerful parallel nature of microarrays could allow the determination of binding constants, catalytic activity and other important protein parameters of all proteins in the cells, making it possible to elucidate protein function [61]. Since biological functions are carried out primarily by proteins rather than nucleic acids, protein microarray technology could be used to screen parasite antigenic properties and possibly proteins associated with the immune response expressed under particular pathological states. Furthermore, it has been shown that RNA expression levels do not always correlate with protein expression levels, and it is almost impossible to predict biochemical properties of a protein encoded by a given gene simply based on its expression profiles. Protein microarrays could therefore help bridge this gap by providing data on which mRNAs are further translated to proteins during filarial infections. Protein microarrays have proven useful in the detection and analysis of various types of protein-ligand interactions, such as protein-protein, protein-DNA, protein-RNA, protein-lipid, protein-drug, and protein-glycan interactions [62,63,64,65,66,67,68,69,70,71], and in the identification of substrates of various classes of enzymes, such as protein kinase, ubiquitin E3 ligase, and acetyltransferase [72,73,74,75,76].

Unfortunately, there are some hitches as far as protein microarrays are concerned. When compared to nucleic acids, proteins have quite diverse and complex biochemical properties, making both their handling and manipulations prior to and during analysis, at times, challenging. Furthermore, the amplification of proteins to generate multiple copies for analysis poses another huge challenge as there are no ready methods for protein amplification compared to the conventional methods such as PCR for nucleic acids. In addition, many proteins are liable to denaturation or degradation in standard buffer conditions and ambient temperatures, rendering protein analysis a bit tedious [77]. While most researchers in the field of filarial infections prefer the use of transcript expression profiles in identifying regulated genes and pathways, others are beginning to opt for protein-based microarrays given the fact that the latter provides more insight into host-parasite interaction. In addition, protein microarrays have the ability of enhancing the current understanding of filarial immunobiology and could also be useful in identifying parasite-induced proteins, which point to protective immune responses.

While exploring other branches of microarrays, it is important to consider recent innovative technologies such as Roche NimbleGen and PepperPRINT, which can achieve high-throughput transcript and peptide expression profiles, respectively. However, a search on PubMed to compare the application of these technologies in human filarial research yielded no results. This is not uncommon given the fact that they are relatively new technologies, and might therefore take some time before becoming the mainstay techniques in filarial research. Nevertheless, we have great expectations for protein microarrays and would emphasize that human filarial research stands in the best position of benefiting from these technologies should they be put in use, particularly in the area of vaccine development and drug design.

## 7. Trends of Microarray Technologies over a 10-Year Period

**Figure 3:**
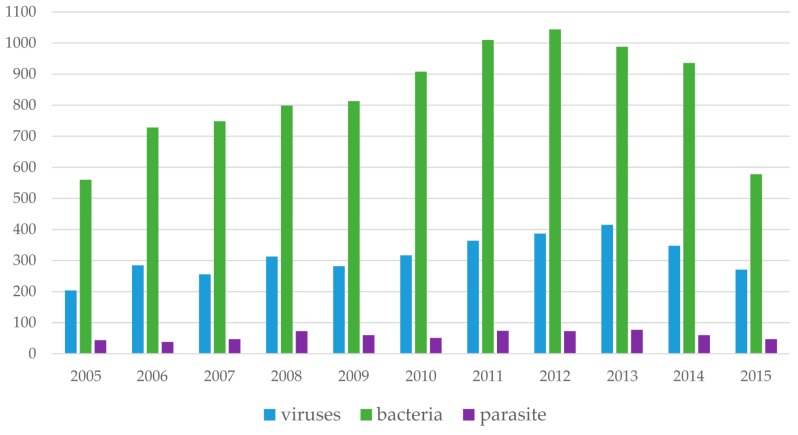
Citations of “Microarray AND Viruses”, “ Microarray AND Bacteria”, and “Microarray AND Parasite” (2005–2015). (The search term “Microarray AND Viruses”, “Microarray AND Bacteria”, and “Microarray AND Parasite” was used to retrieve peer-reviewed research articles published between 2005 and 2015. Endnote X7 was used to query all fields of the PubMed database Refer to main text for search terms and database searched).

**Figure 4:**
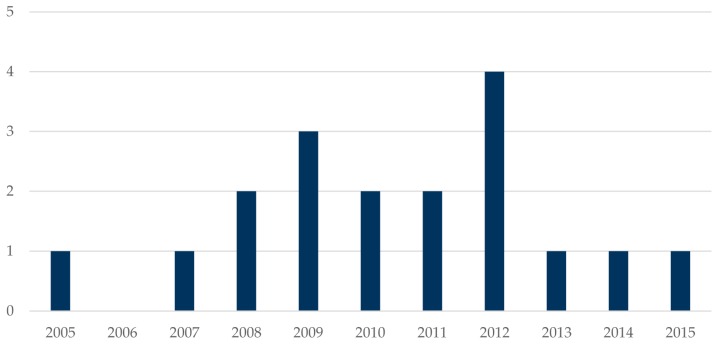
Citations of “Microarray AND Filarial” (2005–2015). The search term “Microarray AND Filarial” was used to retrieve peer-reviewed research articles published between 2005 and 2015. Endnote X7 was used to query all fields of the PubMed database.

This section presents a cursory review of the trend of microarray-based technologies over the past decade. PubMed was queried using the citation manager Endnote X7. The keywords “Microarray AND Bacteria” “Microarray AND Virus” and “Microarray AND Parasite” were used, while varying the period from 2005 to 2015. The search yielded 13,198 publications in all. Interestingly, there was an avalanche of publications referring to microarrays and all pathogen research (bacteria, virus, and parasite) beginning from the year 2009 to 2014 (Figure 3). Perhaps this could be due to the increasing popularity coupled with the drastic reduction in the initial cost of microarrays over the years. While transcriptome profiling of bacteria has been widely exploited, that of filarial research seems to be neglected, which is not surprising considering it has been classified as a neglected tropical disease. Again, the high number of bacteria-microarray publications could be due to the more diverse and ubiquitous nature of bacteria and bacterial infections. On the other hand, when the query was refined to recover publications pertaining to “microarray and filarial” infections, only 18 publications in total were retrieved (Figure 4), providing clear evidence of the novelty of microarray-based technologies in filarial research. In contrast, we found 264 publications in PubMed when the search “Microarray AND Malaria” was performed. This indicates that the technology is commonly used in malaria compared to filarial infection.

## 8. The Future of Microarray in Developing Countries

Most developing countries are the hub of several infectious diseases, accounting for the low quality of life and an entrenched cycle of poverty. There is, therefore, a need for better health systems and research into common and neglected diseases in these countries. Despite the huge benefits of microarray technologies, little remains to be said about Africa and other developing countries. At the moment, there are only a few well-equipped microarray centers in developing countries, though some researchers have collaborations with partners in developed countries working on microarray-based experiments. One major bottleneck in implementing microarray-based research in most developing countries has been the economics of the technology and its related cost. Researchers in most developing countries receive very little governmental and institutional support towards basic research. Hence, the huge cost of the technology discourages the adoption of microarray-based technologies. Another hindrance to the implementation of microarray-based experiments is the lack of expertise in developing countries. Encouraging scientific collaborations which promote the transfer of technology, skills and personnel across continents would be key to implementing microarray experiments in developing countries. In addition, there is a need for various governments to prioritize research on Neglected Tropical Diseases (NTDs). Fostering strong partnerships between academia and industry can also go a long way in promoting microarray-based research in these countries.

## 9. Conclusions

This paper reviewed expression microarrays for use in host response in nematode infections, and how best to use whole blood for transcriptome profiling. Although expression microarrays have proliferated, there still exist unexploited technologies to aid in the investigation of pathogenesis; glycan, protein, and lipid microarrays are valid and underutilized tools for the determination of antigenic properties, mechanisms, and pathophysiology of infections in addition to basic understanding of the biology of nematode parasites. Finally, integration of microarray technology in human filarial nematodes and other neglected tropical diseases may offer insights into novel vaccine candidates, drug targets and other treatment interventions. These techniques require highly skilled personnel and well equipped laboratories, which most often is a challenge in developing countries, Ghana being no exception.

## Abbreviations

The following abbreviations are used in this manuscript:

CFA	Circulating Filarial Antigen
MF	Microfilaria
PCR	Polymerase Chain Reaction
miRNA	Micro-Ribonucleic acid
NGS	Next Generation Sequencing
